# Association of plasma ceramide with stroke-associated pneumonia in acute ischemic stroke

**DOI:** 10.3389/fimmu.2026.1739949

**Published:** 2026-03-04

**Authors:** Hao Li, Mengwen Lu, Xinyi Ye, Xinyan Guo, Tianyu Ma, Zhouao Zhang, Mingjin Yang, Xue Du, Yingying Wang, Xiaoyu Huang, Ying Sheng, Yu Bi, Yong Zhang

**Affiliations:** 1 Department of Neurology, The Affiliated Hospital of Xuzhou Medical University, Xuzhou, Jiangsu, China; 2 Department of Neurology, The Second Affiliated Hospital of Xuzhou Medical University, Xuzhou, Jiangsu, China; 3 Department of Neurology, Suzhou Ninth People’s Hospital, Suzhou, Jiangsu, China; 4 School of Medicine, Tongji University, Shanghai, China; 5 Department of Neurology, Fengxian People’s Hospital, Xuzhou, Jiangsu, China

**Keywords:** biomarker, ceramide, ischemic stroke, propensity score matching (PSM), stroke-associated pneumonia

## Abstract

**Background:**

Stroke-associated pneumonia (SAP) is a common and severe complication of acute ischemic stroke (AIS), yet its occurrence remains unpredictable. Post-stroke immune dysregulation and systemic inflammatory responses play a crucial role in susceptibility to SAP, highlighting the need for immune-related biomarkers. Ceramide (Cer) is kind of bioactive sphingolipids involved in inflammatory signaling and immune cell regulation, and has been implicated in infection and inflammatory diseases. This study aims to explore the association between Cer and SAP and evaluate the predictive value for SAP occurrence.

**Methods:**

This study retrospectively collected a total of 266 eligible patients with AIS and 93 healthy controls. Demographic and clinical data, as well as the concentrations of plasma C16:0-Cer, C18:0-Cer, C24:1-Cer, and C24:0-Cer, were obtained from medical records and were compared before and after propensity score matching. The least absolute shrinkage and selection operator (LASSO) regression was utilized to select variables, and risk factors were detected by multivariate analysis. The predictive values were evaluated by receiver operating characteristic curves.

**Results:**

The levels of C16:0-Cer, C18:0-Cer, C24:1-Cer, C24:0-Cer and Coronary Event Risk Test 1 (CERT1) score were higher in patients with AIS than healthy controls, in which C16:0-Cer, C18:0-Cer and CERT1 score significantly elevated in SAP patients compared with non-SAP patients. Patients with minor ischemic stroke had lower levels of C16:0-Cer, C18:0-Cer and CERT1 score with those with moderate and severe ischemic stroke. Meanwhile, levels of C16:0-Cer, C18:0-Cer and CERT1 score were positively correlated with the National Institutes of Health Stroke Scale (NIHSS) and modified Rankin scale (mRS) score. The A2DS2 score, C16:0-Cer, high-sensitivity C-reactive protein, and neutrophil-to-lymphocyte ratio were identified as independent risk factors for SAP. C16:0-Cer exhibited a predictive value with an area under curve of 0.725, sensitivity of 74.0%, and specificity of 61.2% for SAP.

**Conclusion:**

Plasma C16:0-Cer, C18:0-Cer, and CERT1 scores were significantly elevated in patients with AIS and SAP. Among them, C16:0-Cer served as an independent predictor for SAP in AIS patients. It demonstrated moderate predictive accuracy, suggesting its potential as a novel biomarker for early SAP risk stratification.

## Introduction

Acute ischemic stroke (AIS) is the most prevalent form of stroke in China, with an annual age-standardized incidence of approximately 246.8 per 100,000 people and a crude mortality rate of 62.2 per 100,000 people globally (1, 2). Stroke-associated pneumonia (SAP), defined as new lower respiratory tract infections within seven days after stroke, is one of the most frequent and severe complications after AIS and is associated with prolonged hospitalization, poor functional outcomes, and increased mortality (3).

Traditionally, SAP has been attributed to neurological deficits such as disturbance of consciousness, dysphagia, and aspiration. However, more and more evidence indicates that stroke-induced immune dysregulation plays an important role in the development of SAP (4). Acute ischemic stroke rapidly could activate the sympathetic nervous system (SNS) and leads to excessive catecholamine release, which suppresses cellular immunity by inducing peripheral lymphopenia, promoting lymphocyte apoptosis, and impairing monocyte and natural killer cell function (5, 6). Another study using a mouse model of cerebral ischemia, Prass K, et al. demonstrated that stroke induced marked lymphocyte apoptosis, reduced circulating T-cell numbers, and caused a shift from a shift from T helper cell (Th)1 to Th2 dominant immune responses, resulting in increased susceptibility to infection (6). The activation of hypothalamic-pituitary-adrenal (HPA) axis after stroke may further exacerbate immunosuppression by promoting the apoptosis of T cells and inhibiting the production of pro-inflammatory cytokines (7). Furthermore, post-stroke activation of the parasympathetic nervous system releases acetylcholine, which inhibits pro-inflammatory cytokines and reduces the peripheral immune response (8). These pathological mechanisms could cause stroke patients to enter a state of systemic immunosuppression, which is known as the stroke-induced immunosuppression syndrome (SIIS) (9). Several clinical prediction models have been developed to assess the risk of SAP, among which the A2DS2 score is the most widely used and validated (10). These tools relies on demographic data and neurological severity, which may overlook immune and inflammatory mechanisms contributing to infection. Immune associated biomarkers could provide different perspectives on risk prediction and offer deeper pathophysiological insight into SAP.

Ceramide is a class of bioactive sphingolipid that functions as the second messenger of cellular signaling, playing a crucial role in maintaining membrane homeostasis and inflammatory response (11). Dysregulated ceramide metabolism has been implicated in immune dysfunction, chronic inflammation, and increased susceptibility to infectious diseases (12, 13). In humans, ceramides are generated through three major pathways: *de novo* synthesis pathway, sphingomyelin pathway, and salvage pathway (11, 13). Functionally, ceramide can regulate a variety of immune pathological processes, including the migration and activation of neutrophils, the inflammatory response mediated by macrophages, and the production of cytokines. Studies have demonstrated that altered ceramide profiles are involved in inflammatory lung injury, infection related immune responses, and systemic immune dysregulation (14–16). For example, Drobnik W, et al. found that plasma ceramide levels elevated in sepsis patients (17). Also, it has been demonstrated that patients with community-acquired pneumonia expressed noticeably higher C16:0-Cer and C18:0-Cer, which suggested that ceramide may be a novel biomarker for predicting lung infections (18). These mechanisms may suggest that ceramides may contribute to the development of SAP.

Thus, we hypothesized that altered plasma ceramide profiles reflect stroke-induced immune dysregulation and increase the risk of SAP. This study therefore aimed to explore the relationship between plasma ceramides and SAP and assess their potential as immune-related biomarkers for predicting SAP in acute ischemic stroke patients.

## Materials and methods

### Subjects

**Figure 1:**
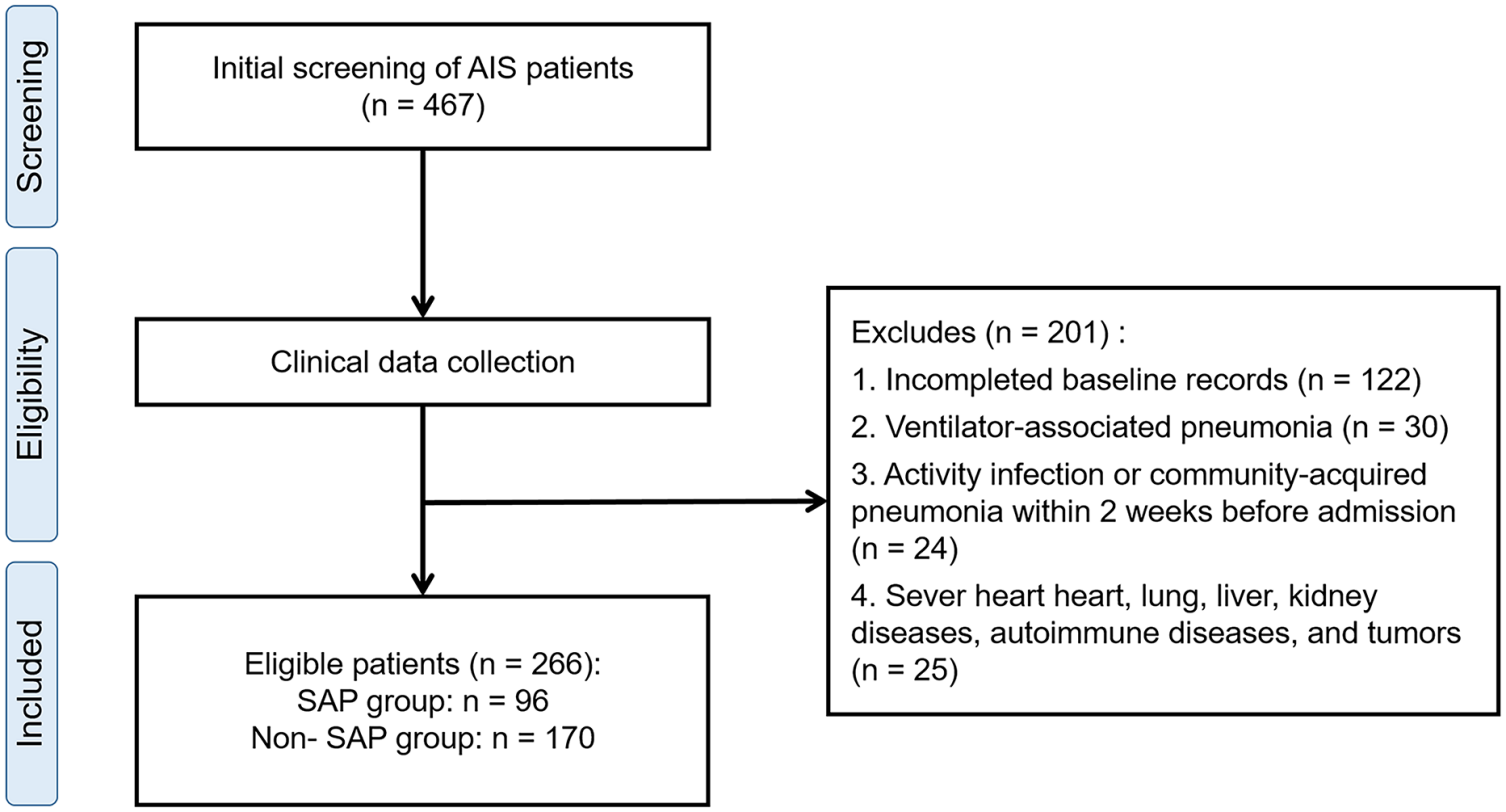
Flow chart of the study. AIS, acute ischemic stroke; SAP, stroke-associated pneumonia.

This was a retrospective observational cohort study. We consecutively collected 467 patients with AIS who have been tested for ceramide levels from the affiliated hospital of Xuzhou Medical University from December 2021 to May 2023. The inclusion criteria were as follows: (1) confirmed diagnosis of AIS based on typical symptoms of neurological deficits and CT scans; (2) hospitalization within 72 hours of symptoms onset. Patients with active infection or any other infection within the past two weeks (n = 24), as well as patients with severe heart diseases (such as New York Heart Association class III-IV heart failure, recent myocardial infarction), lung diseases (such as chronic obstructive pulmonary disease requiring oxygen therapy), liver diseases (such as cirrhosis or liver failure), kidney diseases (such as end-stage renal disease or nephrotic syndrome), autoimmune diseases or malignant tumors (n = 25). In addition, patients with ventilator-associated pneumonia (n=30) or incomplete baseline records (n=122) were eliminated. Finally, 266 patients with completed laboratory tests within 48 hours of admission and baseline records were included and divided into the SAP group (n=96) and the non-SAP group (n=170). The process of selecting patients is provided in **Figure 1**. In addition, 93 age- and sex-matched healthy controls were included from individuals undergoing routine health examinations at our hospital during the study period. All controls had no history of stroke, infection, autoimmune disease, malignant tumor, or chronic inflammatory conditions, and none had a history of smoking or alcohol consumption. A *post hoc* sample size estimation was conducted based on plasma C16:0-ceramide levels. With a significance level (α) of 0.05 and a statistical power (1−β) of 90%, a minimum total of 78 patients was required assuming a SAP-to–non-SAP ratio of 1:2, indicating that the final sample size was adequate for statistical analysis.

### Diagnosis of SAP

The diagnosis of definite SAP was determined according to the criteria established by the Centers for Disease Control and Prevention (3). Diagnosis required the simultaneous fulfillment of systemic, respiratory, and radiological criteria:

(1) Systemic evidence of infection was defined by the presence of at least one of the following: fever (>38 °C) without another explanation; leukocytosis (>12×10^9^/L) or leukopenia (<4×10^9^/L); or, for patients aged ≥70 years, an acute alteration in mental status without another cause. (2) Respiratory manifestations required the presence of at least two of the following: new or exacerbated cough, dyspnea, or tachypnea; increased volume or purulence of respiratory secretions, or a change in sputum character; abnormal findings on lung auscultation; or impaired gas exchange, evidenced by oxygen desaturation or increased oxygen demand. (3) Radiological confirmation was established by chest imaging demonstrating new, progressive, and persistent pulmonary infiltrates, consolidation, or cavitation on at least two serial examinations.

Meanwhile, to avoid confusion with community-acquired pneumonia, all patients underwent routine clinical evaluation at admission to exclude pre-existing pulmonary infection and all the patients with SAP included in this study developed SAP more than 48 hours after admission.

### Data collection

Demographic data, including age, gender and premorbid risk factors containing hypertension, diabetes mellitus, coronary heart disease, atrial fibrillation and history of smoking and stroke, was gathered from medical records. Peripheral venous blood was collected from all patients within 48 hours of hospital admission. In those who later developed SAP, samples were taken before clinical onset to minimize confounding by infection-related inflammation and to evaluate ceramide as early biomarker of SAP. The laboratory results included WBC, neutrophil, lymphocyte, platelet, and albumin levels were collected. Dysphagia was diagnosed at the time of admission with a swallow test. Then, the inflammatory and immune-related indices were selected to reflect different aspects of immune status after stroke. High-sensitivity C-reactive protein (hs-CRP) was used as a marker of systemic inflammatory activation, whereas the neutrophil-to-lymphocyte ratio (NLR) reflects the imbalance between innate immune activation and adaptive immune suppression (19, 20). The platelet-to-lymphocyte ratio (PLR) and the systemic immune-inflammation index (SII), which integrate platelet, neutrophil, and lymphocyte counts, have been proposed as composite indicators of immune–inflammatory status and infection susceptibility in patients with acute ischemic stroke and stroke-associated pneumonia (20, 21). The severity of AIS was assessed by the National Institutes of Health Stroke Score (NIHSS) and modified Rankin scale (mRS). A2DS2 score was calculated (age ≥75 years=1, male=1, AF = 1, dysphagia=2, NIHSS scores: 0-4 = 0, 5-1 = 3, ≥16 = 5) at admission (10). All measurements of laboratory examination were performed by professional laboratory technicians in our hospital.

### Plasma ceramide measurement

Venous blood was collected from on empty stomach at early morning and plasma was separated by centrifugation at 3000 rpm for 10 minutes at 4 °C.

For sample preparation, 100 µL of plasma was combined with 100 µL of internal standard (IS) working solution and 400 µL of ethyl acetate:isopropanol (1:4, v/v). Calibration standards were prepared similarly by replacing plasma with standard solution. After vortexing for 5 minutes, the mixture was centrifuged at 11,000 rpm for 10 minutes at 4 °C. A 300 µL aliquot of the supernatant was recentrifuged under the same conditions, and 100 µL of the resulting supernatant was used for analysis.

High performance liquid chromatography-tandem mass spectrometry (HPLC-MS/MS) analysis was performed on a triple quadrupole mass spectrometer (HPLC-MS/MS, Waters TQD Mass Detector LC/MS/MS, United States) with an electrospray ionization ion source in positive mode. Chromatographic separation was performed on an ACQUITY UPLC BEH C18 column (2.1 mm × 50 mm, 1.7 µm) using a Waters TQD triple quadrupole mass spectrometer equipped with an electrospray ionization source in positive mode. The mobile phases consisted of (A) water containing 1% formic acid and 5 mmol/L ammonium acetate, and (B) isopropanol:acetonitrile (4:3, v/v) with 5 mmol/L ammonium acetate. The flow rate was 0.3 mL/min, and the injection volume was 15 µL. Ceramides were quantified via multiple reaction monitoring (MRM).

### Statistical analysis

Statistical Package for the Social Sciences 26.0 (SPSS 26.0), GraphPad Prism software 9, and R statistical software (version 4.5.0) were utilized for statistical analysis and making figures. Results were presented as percentages for categorical variables and median (interquartile ranges, IQR) for continuous variables. Mann–Whitney U test (abnormal distribution) was utilized to compare two independent samples. Categorical variables were compared by the Chi-squared test or Fisher’s exact test. Propensity score matching (PSM) was applied to minimize potential confounding between groups. Propensity scores were estimated using a multivariable logistic regression model incorporating age, sex, and medical history (hypertension, diabetes, coronary heart disease, stroke, smoking, and atrial fibrillation). Patients were matched using 1:1 nearest-neighbor matching without replacement and covariate balance before and after matching was assessed using standardized mean differences (SMDs). The least absolute shrinkage and selection operator (LASSO) regression analysis was operated with the “glmnet” package. Multivariable logistic regression analysis was utilized to identify the risk factors of SAP. ROC curves were conducted to evaluate the predictive value. The statistical significance was considered as P < 0.05 (two-tailed).

## Results

### Elevated plasma ceramide in patients with acute ischemic stroke

To identify the abnormal expression of ceramide in AIS, plasma ceramide levels were compared between AIS patients and healthy controls. A total of 266 eligible patients with AIS were included into analysis, with a median age of 66.0 (57.0, 72.0) years, a median NIHSS score of 4.5 (2.0, 10.0), and a median mRS score 3.0 (2.0, 4.0). Meanwhile, 93 age-and-sex matched healthy controls were included. There was no statistical difference in age and sex between two groups (all P>0.05, **Table 1**). Significantly, the levels of C16:0-Cer, C18:0-Cer, C24:1-Cer, C24:0-Cer, and the CERT1 score were higher in patients with AIS comparing with healthy controls (all P<0.05, **Table 1**).

**Table 1:** Comparison of baseline characteristics and ceramides levels between healthy controls and patients with acute ischemic stroke.

Variables	Healthy controls (n=93)	AIS patients (n=266)	Effect size	*z/χ^2^*	P-value
Age (years)	63.0 (57.0, 71.5)	66.0 (57.0, 72.0)	0.072	1.014	0.310
Sex, male, n (%)	59 (63.4)	175 (65.7)	0.022	0.167	0.682
C16:0-Cer (μmol/L)	0.33 (0.26, 0.42)	0.47 (0.38, 0.60)	0.689	6.935	**<0.001**
C18:0-Cer (μmol/L)	0.05 (0.04, 0.07)	0.09 (0.05, 0.14)	0.613	7.151	**<0.001**
C24:1-Cer (μmol/L)	0.84 (0.56, 1.28)	1.28 (0.85, 1.78)	0.571	5.330	**<0.001**
C24:0-Cer (μmol/L)	2.52 (1.80, 4.02)	3.24 (2.11, 4.43)	0.248	2.078	**0.038**
CERT1 score	4.00 (2.50, 6.00)	7.0 (5.0, 8.0)	1.019	7.917	**<0.001**

The effect size for continuous measures was expressed as Cohen’s d, while for categorical data, Cohen’s w was reported. Values in bold indicate statistical significance (P<0.05). AIS, acute ischemic stroke; Cer, ceramide; CERT1, Coronary Event Risk Test 1.

### Association between ceramide and the severity of acute ischemic stroke

To investigate the association between ceramide and the severity of stroke, we divided 266 AIS patients into the minor ischemic stroke (MIS) group (n=149) and the non-MIS group (n=117). MIS was defined as an NIHSS score ≤5-points. The levels of plasma C16:0-Cer, C18:0-Cer, and the CERT1 score were significantly lower in the MIS group compared with those in patients with moderate and severe ischemic stroke (all P<0.001, **Table 2**). The incidence of SAP was higher in the non-MIS group (P<0.001, **Table 2**). Besides, there were significant differences in hypertension, dysphagia, atrial fibrillation history, mRS score, A2DS2 score, WBC count, neutrophil count, lymphocyte count, hs-CRP, albumin, NLR, PLR, and SII between the two groups (all P < 0.05, **Table 2**). To adjust for confounding factors between the two groups, we matched age, gender, and medical history variables (hypertension, diabetes, coronary heart disease, stroke, smoking, and atrial fibrillation) using PSM. A total of 94 patients with MIS were matched 1:1 with 94 non-MIS patients. After matching, most categorical covariates achieved acceptable balance, with SMDs below 0.1, only age remained a mild imbalance, with a SMD of 0.177. The significant results obtained from this matching process were consistent with the findings before matching (**Table 2**).

**Table 2:** Comparison of characteristics between patients in MIS group and non-MIS group before and after propensity score matching.

Variables	Unmatched				Propensity score matching			
	MIS (n=149)	Non-MIS (n=117)	SMD	P-value	MIS (n=94)	Non-MIS (n=94)	SMD	P-value
Age (years)	64.0 (56.0, 71.0)	67.0 (59.0, 74.5)	0.213	**0.029**	63.5 (55.8, 71.0)	67.0 (57.8, 74.2)	0.177	0.117
Sex, male, n (%)	95 (63.8)	80 (68.4)	0.098	0.431	66 (70.2)	63 (67.0)	0.069	0.637
Medical history, n (%)								
Hypertension	85 (57.0)	83 (70.9)	0.293	**0.020**	64 (68.1)	64 (68.1)	0.000	1.000
Diabetes	50 (33.6)	33 (28.2)	0.116	0.350	28 (29.8)	31 (33.0)	0.069	0.637
Coronary heart disease	21 (14.1)	16 (13.8)	0.012	0.922	10 (10.6)	12 (12.8)	0.066	0.650
Stroke	33 (22.1)	38 (32.5)	0.233	0.059	25 (26.6)	27 (28.7)	0.048	0.744
Smoking	42 (28.2)	43 (36.8)	0.184	0.137	29 (30.9)	32 (34.0)	0.068	0.640
Atrial fibrillation	4 (2.7)	18 (15.4)	0.454	**<0.001**	4 (4.2)	3 (3.2)	0.056	0.500
Dysphagia, n (%)	8 (5.4)	84 (71.8)	1.866	**<0.001**	6 (6.4)	65 (69.1)	1.699	**<0.001**
SAP, n (%)	17 (11.4)	77 (65.8)	1.347	**<0.001**	4 (4.2)	61 (64.9)	1.655	**<0.001**
NIHSS score	2.0 (2.0, 3.5)	11.0 (8.0, 18.0)	2.024	**<0.001**	2.0 (2.0, 3.0)	11.0 (8.0, 16.0)	1.822	**<0.001**
mRS score	2.0 (1.0, 2.0)	4.0 (3.5, 5.0)	2.470	**<0.001**	2.0 (1.0, 2.0)	4.0 (3.0, 5.0)	2.502	**<0.001**
C16:0-Cer (μmol/L)	0.41 (0.34, 0.53)	0.55 (0.42, 0.67)	0.521	**<0.001**	0.39 (0.30, 0.51)	0.55 (0.41, 0.68)	0.649	**<0.001**
C18:0-Cer (μmol/L)	0.07 (0.05, 0.12)	0.11 (0.07, 0.16)	0.142	**<0.001**	0.07 (0.05, 0.13)	0.10 (0.07, 0.15)	0.089	**0.002**
C24:1-Cer (μmol/L)	1.21 (0.83, 1.76)	1.36 (0.88, 1.81)	0.056	0.306	1.20 (0.86, 1.70)	1.36 (0.96, 1.83)	0.221	0.225
C24:0-Cer (μmol/L)	3.24 (2.14, 4.76)	3.24 (2.06, 4.09)	0.152	0.489	3.00 (2.07, 4.52)	3.28 (2.00, 4.12)	0.015	0.699
CERT1 score	7.0 (4.0, 8.0)	8.0 (6.0, 10.0)	0.566	**<0.001**	7.0 (4.0, 8.0)	8.0 (5.0, 10.0)	0.522	**0.001**

Values in bold indicate statistical significance (P<0.05). Cer, ceramide; CERT1, Coronary Event Risk Test 1; mRS, modified Rankin scale; MIS, minor ischemic stroke; NIHSS, National Institutes of Health Stroke Score; SMD, standardized mean difference.

Furthermore, Spearman’s correlation analysis revealed statistically significant yet modest positive associations of C16:0-Cer, C18:0-Cer, and CERT1 score with NIHSS score (r=0.299, P<0.001; r=0.305, P<0.001; r=0.304, P<0.001) and mRS score (r=0.256, P<0.001; r=0.230, P<0.001; r=0.244, P<0.001) in AIS patients. However, no meaningful correlation was detected in C24:1-Cer, and C24:0-Cer (all P>0.05).

### Elevated plasma ceramide in patients with stroke-associated pneumonia

The demographic data, clinical data, and ceramide levels before and after PSM were compared between the SAP group (n=96) and the non-SAP group (n=170). Before matching, SAP patients were older than non-SAP patients and had a higher incidence of atrial fibrillation and dysphagia (all P<0.001, **Table 3**). There were significant differences in NIHSS score, mRS score, A2DS2 score, WBC count, neutrophil count, lymphocyte count, hs-CRP, albumin, NLR, PLR and SII between the two groups (all P<0.001, **Table 3**). The levels of C16:0-Cer, C18:0-Cer, and the CERT1 score were significantly higher in patients with SAP compared with those in the non-SAP group (all P<0.001, **Table 3**). However, no difference was detected in C24:1-Cer and C24:0-Cer. After matching for age, gender, and medical history variables, a total of 76 non-SAP patients were matched 1:1 with 76 SAP patients. After matching, most categorical covariates achieved acceptable balance, with SMDs below 0.1, only coronary heart disease history remained a mild imbalance, with a SMD of 0.156. After matching, there were significant differences in the NIHSS score, mRS score, A2DS2 score, WBC count, neutrophil count, lymphocyte count, hs-CRP, albumin, NLR, PLR, and SII between the two groups (all P<0.001, **Table 3**). The levels of C16:0-Cer, C18:0-Cer, and the CERT1 score were still significantly higher in SAP patients (all P<0.05, **Table 3**).

**Table 3:** Comparison of characteristics between patients in non-SAP group and SAP group before and after propensity score matching.

Variables	Unmatched				Propensity score matching			
	Non-SAP (n=170)	SAP (n=96)	SMD	P-value	Non-SAP (n=76)	SAP (n=76)	SMD	P-value
Age (years)	63.0 (55.0, 70.2)	69.5 (63.0, 76.0)	0.493	**<0.001**	67.0 (58.2, 74.8)	68.0 (62.0, 73.0)	0.022	0.847
Sex, male, n (%)	105 (61.8)	70 (72.9)	0.239	0.066	57 (75.0)	54 (71.0)	0.089	0.584
Medical history, n (%)								
Hypertension	102 (60.0)	66 (68.8)	0.183	0.155	46 (60.5)	49 (64.5)	0.082	0.615
Diabetes	55 (32.4)	28 (29.2)	0.069	0.590	25 (32.9)	26 (34.2)	0.028	0.864
Coronary heart disease	20 (11.8)	17 (17.7)	0.168	0.179	8 (10.5)	12 (15.8)	0.156	0.337
Stroke	41 (24.1)	30 (31.3)	0.160	0.207	23 (30.3)	20 (26.3)	0.088	0.589
Smoking	48 (28.2)	37 (38.5)	0.220	0.083	26 (34.2)	28 (36.8)	0.055	0.735
Atrial fibrillation	6 (3.5)	16 (16.7)	0.447	**<0.001**	6 (7.9)	6 (7.9)	0.000	1.000
Dysphagia, n (%)	22 (12.9)	70 (72.9)	1.523	**<0.001**	12 (15.8)	54 (71.0)	1.343	**<0.001**
NIHSS score	3.0 (2.0, 5.0)	12.0 (7.0, 19.0)	1.511	**<0.001**	3.5 (2.0, 6.8)	11.0 (6.0, 16.0)	1.129	**<0.001**
mRS score	2.0 (1.0, 3.0)	4.0 (3.0, 5.0)	1.293	**<0.001**	2.0 (2.0, 3.0)	4.0 (3.0, 5.0)	0.962	**<0.001**
A2DS2 score	1.0 (1.0, 3.0)	6.0 (5.0, 8.0)	1.782	**<0.001**	2.0 (1.0, 4.0)	6.0 (4.0, 7.0)	1.226	**<0.001**
WBC (10^9^/L)	6.15 (5.10, 7.60)	9.10 (7.12, 10.90)	1.189	**<0.001**	6.15 (5.30, 7.68)	9.45 (7.38, 11.10)	1.064	**<0.001**
Neutrophil (10^9^/L)	3.84 (3.00, 5.07)	7.23 (5.46, 9.23)	1.452	**<0.001**	3.78 (3.11, 5.14)	7.46 (5.68, 9.27)	1.282	**<0.001**
Lymphocyte (10^9^/L)	1.70 (1.30, 2.10)	1.00 (0.72, 1.50)	0.766	**<0.001**	1.65 (1.23, 2.00)	1.10 (0.80, 1.50)	0.763	**<0.001**
Platelet (10^9^/L)	215.5 (173.0, 257.0)	201.0 (168.5, 247.8)	0.116	0.148	208.0 (163.8, 249.2)	202.0 (170.0, 252.5)	0.016	0.974
NLR	2.3 (1.8, 3.1)	6.8 (4.6, 11.2)	1.417	**<0.001**	2.4 (1.8, 3.3)	6.6 (4.6, 10.4)	1.136	**<0.001**
PLR	126.5 (102.9, 167.5)	191.9 (122.1, 286.4)	0.882	**<0.001**	129.8 (93.9, 166.9)	184.3 (119.6, 264.4)	0.740	**<0.001**
SII	487.7 (363.7, 704.4)	1484.5 (713.5, 2417.7)	1.143	**<0.001**	501.3 (365.4, 741.2)	1414.7 (704.7, 2123.6)	0.872	**<0.001**
hs-CRP (mg/L)	0.8 (0.5, 2.8)	9.2 (2.0, 45.4)	1.006	**<0.001**	1.0 (0.5, 4.2)	15.2 (2.2, 54.5)	0.897	**<0.001**
Albumin (g/L)	41.7 (39.6, 44.3)	38.2 (34.9, 41.8)	0.847	**<0.001**	41.4 (39.3, 44.4)	38.2 (34.3, 41.98)	0.690	**<0.001**
C16:0-Cer (μmol/L)	0.42 (0.33, 0.55)	0.56 (0.46, 0.68)	0.683	**<0.001**	0.40 (0.31, 0.52)	0.56 (0.45, 0.68)	0.783	**<0.001**
C18:0-Cer (μmol/L)	0.07 (0.05, 0.12)	0.11 (0.07, 0.18)	0.186	**<0.001**	0.07 (0.05, 0.14)	0.10 (0.07, 0.17)	0.020	**0.015**
C24:1-Cer (μmol/L)	1.22 (0.83, 1.75)	1.36 (0.93, 1.84)	0.035	0.441	1.21 (0.84, 1.75)	1.34 (0.92, 1.90)	0.044	0.493
C24:0-Cer (μmol/L)	3.29 (2.11, 4.74)	3.22 (2.15, 3.83)	0.234	0.295	3.02 (1.99, 4.52)	3.26 (2.20, 4.08)	0.042	0.735
CERT1 score	6.0 (4.0, 8.0)	8.0 (7.0, 10.0)	0.691	**<0.001**	6.0 (4.0, 9.0)	8.0 (6.2, 10.0)	0.574	**0.001**

Values in bold indicate statistical significance (P<0.05). Cer, ceramide; CERT1, Coronary Event Risk Test 1; hs-CRP, high-sensitivity C-reactive protein; mRS, modified Rankin scale; NIHSS, National Institutes of Health Stroke Score; NLR, neutrophil-tolymphocyte ratio; PLR, platelet-to-lymphocyte ratio; SAP, stroke associated pneumonia; SII, systemic immune-inflammation index; SMD, standardized mean difference; WBC, white blood cell.

### Predictive value of ceramide for stroke-associated pneumonia

**Figure 2:**
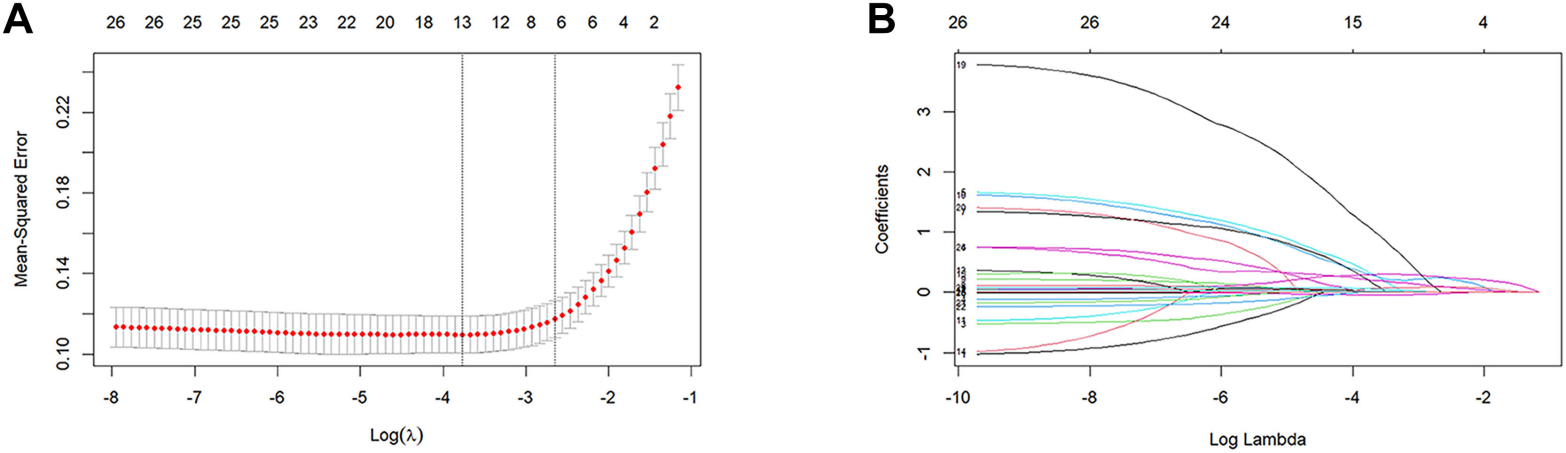
LASSO regression for variable selection. **(A)** The partial likelihood deviance (binomial deviance) curve was plotted versus the log (lambda); **(B)** LASSO coefficient profiles for clinical features, each coefficient profile plot is produced vs log lambda sequence. Dotted vertical lines were drawn at the optimal values by using the minimum criteria and the 1-SE of the minimum criteria to obtain the included feature variables, where nine nonzero coefficients were included.

**Figure 3:**
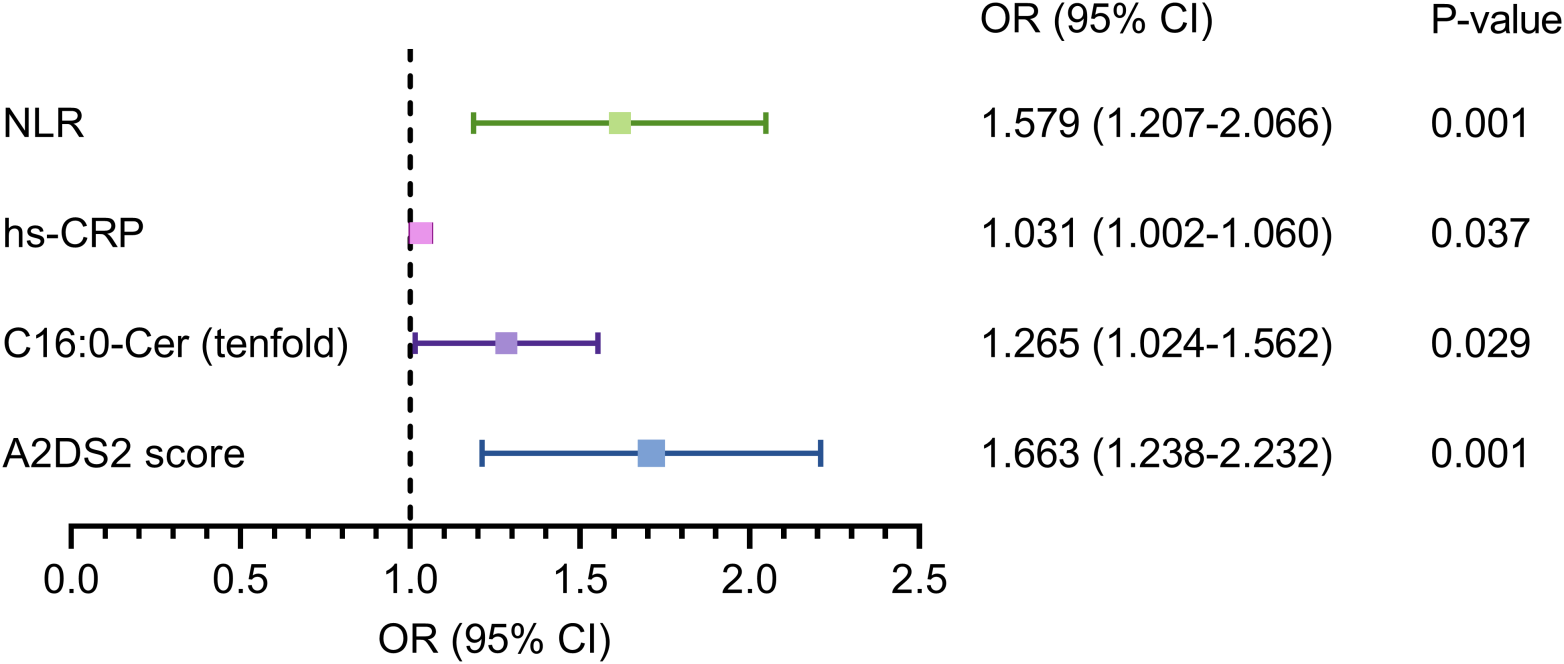
Forest plot for the results of multivariate regression analysis. Cer, ceramide; CI, confidence interval; hs-CRP, high-sensitivity C-reactive protein; NLR, neutrophil-to-lymphocyte ratio.

**Figure 4:**
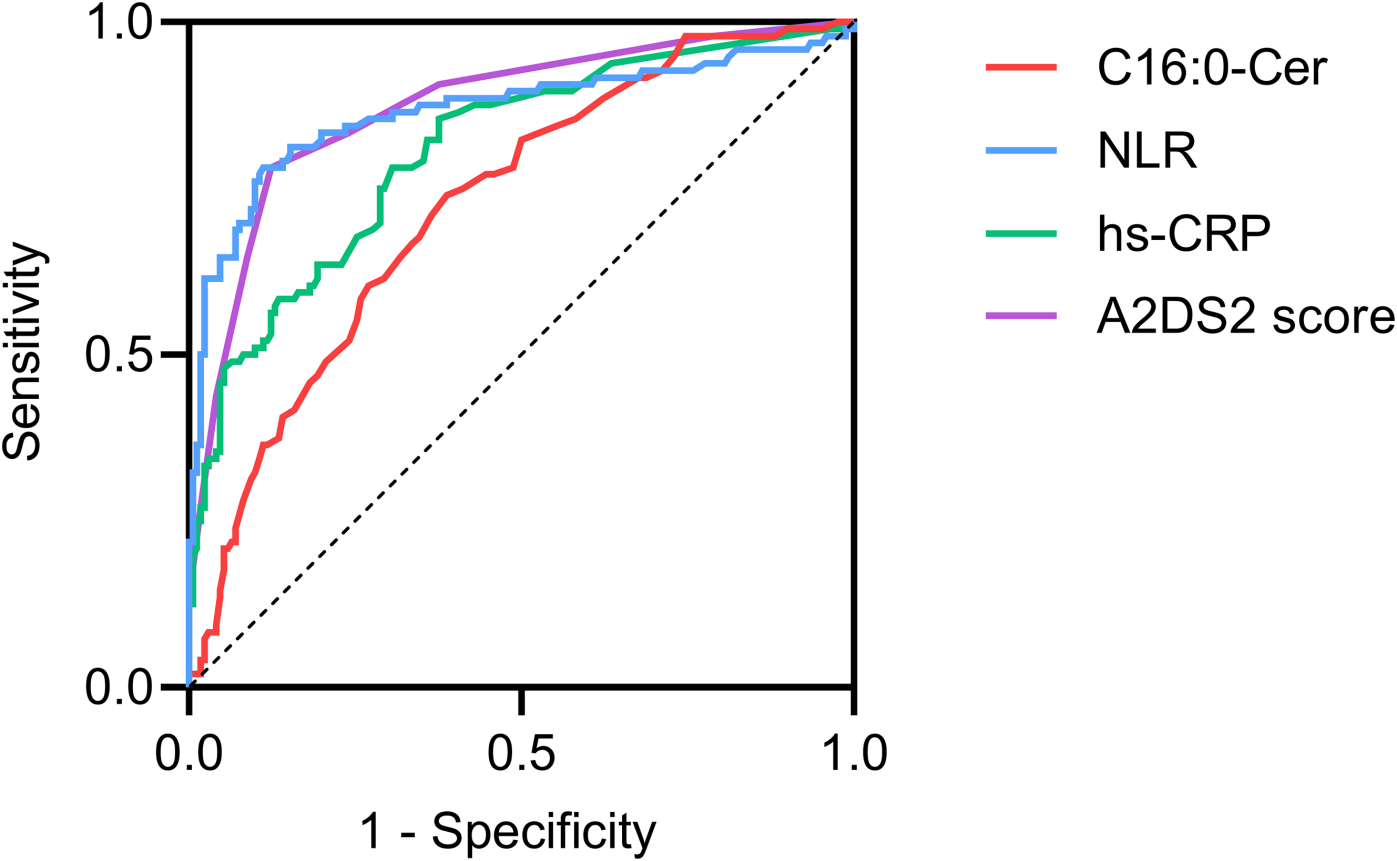
ROC curve was used to evaluate the accuracy of C16:0-Cer, A2DS2 score, hs-CRP, and NLR to predict SAP. Cer, ceramide; hs-CRP, high-sensitivity C-reactive protein; NLR, neutrophil-to-lymphocyte ratio; SAP, stroke-associated pneumonia.

The LASSO regression was performed on all 26 variables, and eight variables (dysphagia, A2DS2 score, neutrophil count, hs-CRP, albumin, NLR, C16:0-Cer, and CERT1 score) that had non-zero coefficients were chosen (**Figures 2A, B**). The eight variables were included in the multivariate regression analysis, and the results were displayed in a forest plot. The A2DS2 score, hs-CRP, C16:0-Cer, and NLR were identified as being independently associated with SAP after stroke (**Figure 3**). ROC curve analysis revealed that C16:0−Cer exhibited a moderate ability to discriminate SAP (AUC: 0.725; 95% CI: 0.664–0.787; P<0.001). However, its predictive performance did not exceed that of established markers: the A2DS2 score (AUC: 0.873; 95% CI: 0.827–0.919; P<0.001), hs−CRP (AUC: 0.807; 95% CI: 0.752–0.862; P<0.001), and NLR (AUC: 0.866; 95% CI: 0.813–0.920; P<0.001) (**Figure 4**). This suggests C16:0-Cer may serve as a complementary biomarker rather than a replacement. The optimal cut-off values, determined by maximizing the Youden index, were 4.5 points for the A2DS2 score, 0.465 μmol/L for C16:0−Cer, 1.25 mg/L for hs−CRP, and 4.3 for NLR, yielding specificities of 0.876, 0.612, 0.624, and 0.888, and sensitivities of 0.781, 0.740, 0.854, and 0.781, respectively.

## Discussion

This study demonstrated that plasma ceramide (C16:0-Cer, C18:0-Cer), and CERT1 score were elevated in AIS and were related to stroke severity. Importantly, patients who developed SAP exhibited higher C16:0-Cer and C18:0-Cer levels, as well as higher CERT1 score; however, after adjustment for clinical and inflammatory factors, only C16:0-Cer remained independently associated with SAP, yet its discriminative ability for SAP was moderate. These findings suggest that C16:0-Cer may serve as a complementary biomarker for identifying patients at increased risk of SAP.

As structural and functional molecules, sphingolipids are important components of the cell membrane and are involved in cell signal transmission (22). Ceramide was considered the center of sphingolipid metabolism (23). Ceramides have pro-inflammatory and pro-aggregatory effects on the vascular endothelium. For example, chronic accumulation of ceramide can trigger reactive oxygen species (ROS) production, which then promotes the activation of pro-oxidative stress pathways, leading to endothelial dysfunction (24, 25). Vascular endothelial injury is closely related to atherosclerosis and stroke. It has also been found that ceramide is abnormally expressed in patients with ischemic stroke and is associated with disease severity and poor prognosis (26, 27). In our study, we similarly found that abnormally elevated C16:0-Cer, C18:0-Cer, C24:1-Cer, C24:0-Cer and CERT1 score in patients with ischemia stroke. Besides, C16:0-Cer, C18:0-Cer, and CERT1 score were higher in moderate and severe stroke patients.

Ceramides may also participate in the development of SAP through modulation of post-stroke immune responses. Stroke is known to induce profound immune dysregulation characterized by early systemic inflammation followed by immunosuppression, increasing susceptibility to infection. Ceramides have been shown to regulate innate immune processes, including neutrophil migration, endothelial transmigration, and chemotaxis, which are critical components of pulmonary host defense (28, 29). In addition, ceramides could influence macrophage activation, cytokine production, and metabolic reprogramming during infection (16, 30). For example, study have shown that elevated ceramide generation is associated with increased production and secretion of pro-inflammatory cytokines such as interleukin (IL)-6, tumor necrosis factor (TNF)-α, and monocyte chemoattractant protein-1 (MCP)-1 in multiple cell models, including adipocytes and macrophages, supporting a role for ceramide in inflammatory signaling pathways relevant to infection and systemic immune responses (31). In clinical settings, specific ceramide species have been found to correlate with higher circulating levels of pro-inflammatory cytokines in patients with cardiovascular disease, indicating that ceramide dysregulation may contribute to systemic inflammatory burdens (32). In turn, TNF-α and IL-1β themselves can enhance sphingomyelin hydrolysis and ceramide production, suggesting a bidirectional interaction between ceramide metabolism and cytokine networks (33). In addition, in clinical cohorts, including the Corogene and SPUM-ACS studies, C16:0-Cer and C18:0-Cer were positively associated with C-reactive protein, supporting a close relationship between ceramides and systemic inflammatory activity (34).

Furthermore, sphingomyelin metabolism and increased expression of ceramide were closely related to the pathological inflammatory response in lung (35). Pseudomonas aeruginosa infection has been shown to induce the activation of acid sphingomyelinase (ASM), leading to the generation of ceramide (36). Meanwhile, Peng H et al. demonstrated that downregulation of ASM and subsequent reduction of ceramide attenuated pulmonary edema in a murine model of Staphylococcus aureus infection (37). These may suggest that ceramide and sphingomyelin metabolism could be a new therapeutic target for bacterial pneumonia. However, any therapeutic implications in the context of SAP should be considered premature. Arshad H et al. reported increased ceramide synthesis and acid sphingomyelinase activity in patients with community-acquired pneumonia (CAP). Their findings demonstrated elevated levels of C16:0-Cer, C18:0-Cer, and C24:1-Cer in these patients, which effectively distinguished them from controls (18). In line with these observations, SAP patients in our study exhibited higher levels of C16:0-Cer and C18:0-Cer compared with non-SAP patients. Notably, only C16:0-Cer showed an independent association with SAP. This finding may be partly explained by the high expression of ceramide synthase 5 (CerS5), the primary enzyme responsible for C16-Cer production, in pulmonary epithelial cells (38). Although the discriminatory performance of C16:0-Cer was inferior to that of established clinical and inflammatory markers, its significant association with SAP highlights a previously underexplored link between sphingolipid metabolism and post-stroke immune dysregulation. This observation supports a potential role for ceramides as complementary indicators of inflammatory biomarkers.

Our study had several limitations. First, this single-center, retrospective study may still be subject to residual confounding, despite the use of multivariate adjustment and propensity score matching. Meanwhile, the mismatch in the severity of stroke between groups may also affect the judgment of C16:0-Cer as an independent factor. Furthermore, the exclusion of some patients due to incomplete data may have introduced additional selection bias into the study population. Second, cytokines and other immune mediators were not directly measured, limiting mechanistic insight into ceramide and cytokine interactions. Third, ceramide levels were assessed at a single time point, precluding evaluation of dynamic changes during SAP development. Finally, external validation in independent cohorts is lacking. Future prospective, multicenter studies incorporating longitudinal sampling and comprehensive immune profiling are warranted to clarify the role of ceramides in post-stroke immune dysregulation and SAP.

## Conclusion

In conclusion, this study provided initial evidence that plasma ceramide profiles change in patients with acute ischemic stroke and correlate with stroke severity and the risk of SAP. Elevated C16:0-Cer levels were linked to higher SAP risk, indicating that it may reflect immune and inflammatory dysregulation after stroke. Thus, C16:0-Cer has a potential serve as a complementary and soluble biomarker, offering additional immunometabolic insight. Due to the observational nature of this study, further studies with external validation, cytokine profiling, and *in vitro* experiment are still needed to clarify the underlying mechanisms and assess whether ceramides, combined with clinical scores and inflammatory markers, can improve SAP risk stratification in a clinically useful way.

## Data Availability

The original contributions presented in the study are included in the article/supplementary material. Further inquiries can be directed to the corresponding authors.
